# Novel engineered IL-2 Nemvaleukin alfa combined with PD1 checkpoint blockade enhances the systemic anti-tumor responses of radiation therapy

**DOI:** 10.1186/s13046-024-03165-x

**Published:** 2024-09-02

**Authors:** Kewen He, Nahum Puebla-Osorio, Hampartsoum B. Barsoumian, Duygu Sezen, Zahid Rafiq, Thomas S. Riad, Yun Hu, Ailing Huang, Tiffany A. Voss, Claudia S. Kettlun Leyton, Lily Jae Schuda, Ethan Hsu, Joshua Heiber, Maria-Angelica Cortez, James W. Welsh

**Affiliations:** 1 Department of Radiation Oncology, Shandong First Medical University and Shandong Academy of Medical Sciences, Shandong Cancer Hospital and Institute, Jinan, Shandong China; 2 Department of Radiation Oncology, Division of Radiation Oncology, The University of Texas MD Anderson Cancer Center, Houston, TX United States; 3 Department of Radiation Oncology, Koç University School of Medicine, Istanbul, Turkey; 4 Mural Oncology PLC, Waltham, MA United States

**Keywords:** Cancer, Radiation, RDB 1462, IL-2, Abscopal, And PD-1

## Abstract

**Background:**

Combining interleukin-2 (IL-2) with radiotherapy (RT) and immune checkpoint blockade (ICB) has emerged as a promising approach to address ICB resistance. However, conventional IL-2 cytokine therapy faces constraints owing to its brief half-life and adverse effects. RDB 1462, the mouse ortholog of Nemvaleukin alfa, is an engineered IL-2 with an intermediate affinity that selectively stimulates antitumor CD8 T and NK cells while limiting regulatory T cell expansion. This study aimed to evaluate the antitumor activity and mechanism of action of the combination of RDB 1462, RT, and anti-PD1 in mouse tumor models.

**Methods:**

Two bilateral lung adenocarcinoma murine models were established using 344SQ-Parental and 344SQ anti-PD1-resistant cell lines. Primary tumors were treated with RT, and secondary tumors were observed for evidence of abscopal effects. We performed immune phenotyping by flow cytometry, analyzed 770 immune-related genes using NanoString, and performed T cell receptor (TCR) repertoire analysis. Serum pro-inflammatory cytokine markers were analyzed by 23-plex kit.

**Results:**

Compared to native IL-2 (RDB 1475), RDB 1462 demonstrated superior systemic antitumoral responses, attributable, at least in part, to augmented levels of CD4 and CD8 T cells with the latter. Our findings reveal substantial reductions in primary and secondary tumor volumes compared to monotherapy controls, with some variability observed among different dosing schedules of RDB 1462 combined with RT. Blood and tumor tissue-based flow cytometric phenotyping reveals an increase in effector memory CD8 and CD4 T cells and a decrease in immunosuppressive cells accompanied by a significant increase in IL-2, IFN-γ, and GM-CSF levels in the combination group. Transcriptomic profiling and TCR sequencing reveal favorable gene expression and T cell repertoire patterns with the dual combination. Furthermore, integrating anti-PD1 therapy with RT and RDB 1462 further reduced primary and secondary tumor volumes, prolonged survival, and decreased lung metastasis. Observations of immune cell profiles indicated that RT with escalating doses of RDB 1462 significantly reduced tumor growth and increased tumor-specific immune cell populations.

**Conclusion:**

The addition of Nemvaleukin therapy may enhance responses to RT alone and in combination with anti-PD1.

**Graphical Abstract:**

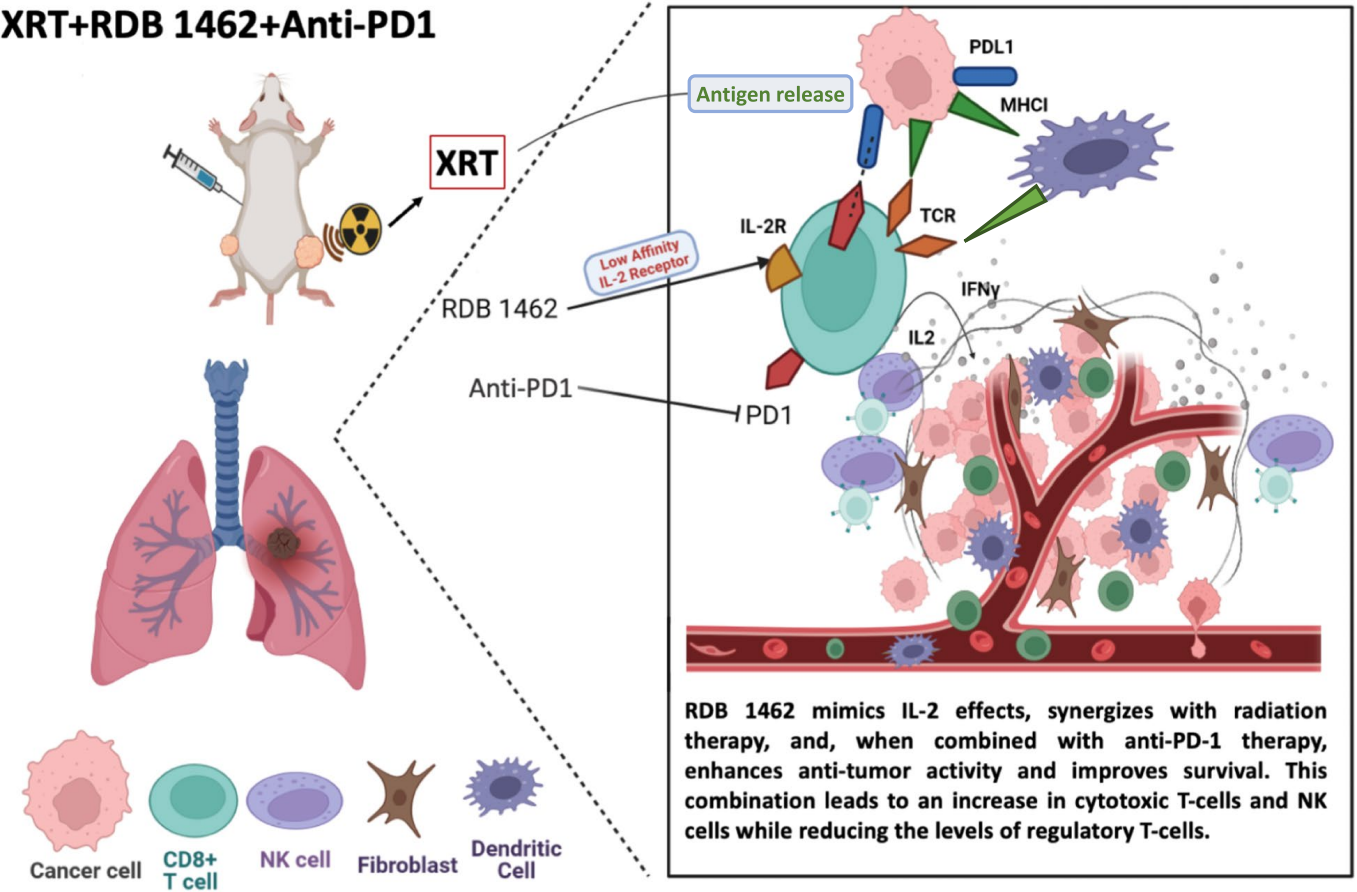

**Supplementary Information:**

The online version contains supplementary material available at 10.1186/s13046-024-03165-x.

## Introduction

Lung cancer stands as the prevailing global malignancy bearing the brunt of cancer-related mortality [1]. While the advent of immune checkpoint blockade (ICB) represents a recent therapeutic breakthrough, its survival benefits remain confined to responsive patients [2]. The synergistic convergence of ICBs with immunogenic radiotherapy (RT) has manifested substantial enhancements in the prognosis of ICB-responsive patients [3]. However, innovative alternatives are necessary, particularly for patients resistant to ICB therapy [4, 5]. Combining interleukin-2 (IL-2) with RT, with or without ICB, presents a potentially transformative approach. In response to antigenic stimulation, effector T cells assume a pivotal role by secreting the IL-2 cytokine, exerting autocrine and paracrine influence over T cells' differentiation, proliferation, and survival. This includes immunosuppressive CD4 + FoxP3 + regulatory T cells (Tregs) and natural killer (NK) cells. Owing to its low molecular weight (15.5 kDa), IL-2 exhibits a brief half-life (t1/2) in circulation [6, 7]. The high-affinity IL-2 receptor, composed of α (CD25), β (CD122), and γ (γc, CD132) chains, is persistently expressed on CD4 + Tregs and transiently in recently activated T cells. In contrast, the intermediate-affinity IL-2 receptor, a dimeric receptor featuring β (CD122) and γc (CD132), is constitutively expressed in memory CD8 + T cells and NK cells [8, 9]. Unfortunately, high-dose IL-2 therapy has shown limited clinical efficacy due to its short half-life, potentially life-threatening adverse events such as cytokine storms, and the induction of immune-suppressive Tregs via binding to its high-affinity receptor [9–11]

Reports indicate that utilizing modified IL-2 that binds to the low-affinity receptor may alleviate these drawbacks. However, comprehensive exploration of the combination of such modified IL-2 with radiotherapy and ICBs remains pending. Nemvaleukin alfa (nemvaleukin), an innovative engineered cytokine, demonstrates selective binding to the intermediate-affinity IL-2 receptor, preferentially stimulating and expanding antitumor CD8 T and NK cells, while minimizing CD4 + regulatory T cell expansion [12]. Thus, Nemvaleukin, whose murine ortholog is RDB 1462, may be beneficial in combination with RT and ICB [13]. This study sought to investigate the impacts of RDB 1462, RT, and immunotherapy on tumor growth, as well as elucidate the underlying mechanisms, including immune cell profiling, within mouse tumor models.

The central concern regarding the safety and tolerability of high-dose IL-2 primarily stems from IL-2's binding to CD25 on vascular endothelial cells, thereby precipitating capillary leak syndrome. Moreover, IL-2 exhibits its antitumor efficacy within a confined subset of patients with antigenic tumor types, such as malignant melanoma or renal cell carcinoma [14, 15]. Both clinical trials referenced employed hypofractionated RT (hRT). Notably, studies have underscored the capacity of moderate or higher-dosed fraction hRT to induce tumor-specific CD8 + cytotoxic T cells [16]. The induction of immunogenic cell death emerges as a pivotal factor, whereby tumor irradiation augments the influx of T cells into tumors. Consequently, the combined application of tumor immunotherapeutics, including ICBs, IL-2, IL-2 variants, and others, with immunogenic RT, promises synergistic potential [11, 16, 17].

In a two-tumor sarcoma mouse model, where only one of the two tumors received irradiation, anti-PD1 was added to the combination of RT and IL-2c (complex, IL-2/anti-IL-2). In this highly radiosensitive model, the control of the irradiated tumor and the levels of CD8 + tumor-infiltrating lymphocytes (TILs) exhibited no significant variance between the RT/IL-2c combination and RT monotherapy groups. Similarly, the control of unirradiated tumors demonstrated no substantial deviation between the RT/IL-2c combination and IL-2c monotherapy groups [9].

In light of these factors, this study sets out to evaluate the antitumor activity and the mechanism of action underpinning the combination of RDB 1462 (the murine counterpart of Nemvaleukin, also known as m Nemvaleukin), RT, and immunotherapy in murine lung adenocarcinoma tumor models.

## Materials and methods

### Cell Lines

The 344SQ-Parental (344SQ-P) cell line, derived from lung adenocarcinoma, was generously provided by Dr. Jonathan Kurie at MD Anderson Cancer Center. It is an aggressively growing cell line with a P53 mutation and KRAS hyperactivation (p53^R172HΔg/+^ K-ras^LA1/+^). We also used a 344SQ anti-PD1-resistant cell line (344SQ-R) that had previously been derived from 344SQ-P cells in our laboratory through selective in vivo passaging under anti-PD1 pressure [18]. Cells were cultured in RPMI-1640 medium supplemented with 100 U/mL penicillin, 100 mg/mL streptomycin, and 10% fetal bovine serum, then incubated at 37 °C in 5% CO_2_.

### Mice and tumor establishment

In this study, male 129 Sv/Ev mice aged between 10 and 12 weeks were selected and bred in-house. These mice were maintained in a controlled environment that was free from pathogens. All experimental procedures involving animals were conducted strictly with the guidelines and regulations established by UT MD Anderson's Institutional Animal Care and Use Committee [19]. Our experimental setup established bilateral tumor models with primary tumors implanted on day 0 and secondary tumors on day 3. To initiate tumor growth, we subcutaneously injected 344SQ-P or 344SQ-R lung adenocarcinoma cells into the hind legs of 129 Sv/Ev mice. We used 0.4 × 10^6^ cells in the right leg for the primary tumors, while for the secondary tumors, we injected 0.1 × 10^6^ cells in the left leg. Throughout the study, the in vivo manipulators measured the tumor size twice weekly using digital calipers and were blinded to the experimental groups. Mice were euthanized if the primary or secondary tumors reached a diameter of 15 mm, following ethical guidelines.

### Treatments and drugs

X-ray radiation therapy (XRT) was employed to treat the primary tumors, administering three fractions of 12 Gy each (total dose of 36 Gy), while the secondary tumors remained untreated. The initiation of XRT occurred on day 7 when the tumors had achieved an average diameter of 7 mm and mice were randomized into different groups to normalize tumor sizes. The in vivo technicians were blinded from the experimental groups and treatments. For drug preparation, RDB 1462, RDB1475 and anti-PD1 (RDB 3911/3907) were all provided by Alkermes. We administered RDB 1462 via subcutaneous (s.c.) injection, following various dosing schedules outlined in the figures. Similarly, RDB 1475 was administered via subcutaneous (s.c.) injections, adhering to different dosing schedules indicated in the figures. Anti-PD1 was given via intraperitoneal (i.p.) injections at a dose of 10 mg/kg, following different schedules outlined in the figures.

### Quantification of lung metastasis

Lungs were harvested at experimental endpoints and stored in Bouin’s fixative solution (HT10132-1L, MilliporeSigma). Using a magnifier lamp, the lung metastases spots were counted.

### Sample processing and Flow cytometric immune phenotyping

Blood from mice (5 mice/group) was drawn from the cheek using anticoagulant tubes (with Heparin, cat. 20.1282.100), and peripheral blood mononuclear cell (PBMC) suspension was obtained by Lymphoprep (cat. 1,114,544). As indicated in the figures, blood samples were collected at designated time points for flow cytometric immune profiling, using the following panels: CD45 Spark NIR 685 (Clone: 30-F11, Cat. 103,168), CD3 BV570 (Clone: 17A2, Cat. 100,225), CD4 PE-CF594 (Clone: GK1.5, Cat. 100,456), CD8 PercpCy5.5 (Clone: 53–6.7, Cat. 100,734), CD25 PE-Cy5 (Clone: PC61, Cat. 102,010), CD44 BV750 (Clone: IM7, Cat. 103,079), CD62L AF700 (Clone: MEL-14, Cat. 104,426), NK1.1 PE-Cy7 (Clone: PK136, Cat. 108,714), CD19 FITC (Clone: 1D3/CD19, Cat. 152,404), Foxp3 PE (Clone: MF-14, Cat. 126,404) for the lymphoid lineage, and CD45 Spark NIR 685 (Clone: 30-F11, Cat. 103,168), CD11b BV421 (Clone: M1/70, Cat. 101,236), granulocyte receptor-1 (Gr1) BV711 (Clone: RB6-8C5, Cat. 108,443) for the myeloid phenotype (all from Bio-Legend). In a separate experiment, 344SQ-P tumors were established bilaterally in 129 Sv/Ev mice, divided into six groups, and subjected to different treatment regimens as outlined in the figure. On day 16, single-cell suspensions from freshly isolated tumor tissues (both primary and secondary tumors, 5 mice/group) were prepared in RPMI 1640 with 250 μg/mL of Liberase TR and 20 μg/mL DNase I and further dissociated with a gentleMACS™ Octo Dissociator with Heaters (MiltenyiBiotec), according to the manufacturer’s protocol, and filtered afterwards. Spleens were mashed against the surface of a 40 μm cell strainer using a plunger of 1 ml syringe. Lymphoprep (cat. 1,114,544) enriched lymphocytes from tumor tissue, while red blood cells were removed from spleens using ACK lysing buffer (Lonza, cat. BP10-548E). The following antibody panel was employed for immune phenotyping: CD45 Spark NIR 685 (Clone: 30-F11, Cat. 103,168), CD3 BV570 (Clone: 17A2, Cat. 100,225), CD4 PE-CF594 (Clone: GK1.5, Cat. 100,456), CD8 PercpCy5.5 (Clone: 53–6.7, Cat. 100,734), CD25 PE-Cy5 (Clone: PC61, Cat. 102,010), CD44 BV750 (Clone: IM7, Cat. 103,079), CD62L AF700 (Clone: MEL-14, Cat. 104,426), NK1.1 PE-Cy7 (Clone: PK136, Cat. 108,714), Foxp3 PE (Clone: MF-14, Cat. 126,404), interferon-gamma (IFN-γ) FITC (Clone: XMG1.2, Cat. 505,806), Ki67 Pacific blue (Clone: 16A8, Cat. 652,422), Fas BV605 (Clone: SA367H8, Cat. 152,612), FasL APC (Clone: MFL3, Cat. 106,610) for the lymphoid lineage (all from Bio-Legend). All samples were processed using the Aurora Flow Cytometer (Cytek Biosciences) at the Flow Cytometry Core Facility at MD Anderson Cancer Center (MDACC) and analyzed with FlowJo V10 software.

### Nanostring

We established bilateral tumors using the 344SQ-P lung adenocarcinoma cells in four separate groups of 129 Sv/Ev mice, with three mice per group. Bilateral tumors using the 344SQ-R cells were also implanted in another four groups. The experimental groups received different treatments, including XRT alone, XRT + RDB 1462, or XRT + RDB 1462 + anti-PD1. On day 16, we collected secondary tumors from all groups for NanoString analysis. Briefly, RNA samples were extracted from tumor-infiltrating lymphocytes, quality checked, and then submitted to the Advanced Technology Genomics Core at MDACC for NanoString analysis. The NanoString nCounter Pan Cancer Immune Panel measures gene expression levels in the collected samples of 770 immune-related genes for expression profiling. This panel allowed us to comprehensively analyze gene expression patterns relevant to immune responses and cancer. The expression profiling was conducted using the nCounter FLEX Instrument, which enabled precise quantification of gene expression levels. The raw NanoString data were analyzed and normalized with nSolver Software v4.0 and nCounter Advanced Analysis (NanoString Technologies) (see detailed information in Supplementary Methods).

### Cytokine profiling

We analyzed serum pro-inflammatory cytokine markers to assess the systemic immune response and the presence of key cytokines associated with inflammation. We employed a 23-plex kit (M60009RDPD, Bio-Rad Laboratories) to measure the levels of various pro-inflammatory cytokines, including tumor necrosis factor alpha (TNF-α), interleukin-6 (IL-6), IFN-γ, interleukin-1 beta (IL-1β), among others, according to the manufacturer’s protocol. Briefly, blood samples were collected from the cheek of 5 mice/ group on day 16 and centrifuged for 20 min at 2000 × g. Sera were collected and then diluted by 1:2 before loading and incubating with detection antibodies and SA-PE. The 96-well plate was read by Bio-Plex MAGPIX System (see detailed information in Supplementary Methods).

### T cell receptor (TCR) repertoire analysis

We use TCR repertoire analysis by next-generation sequence to study the composition and clonal diversity of TCRs within the tumor T cell populations. This analysis provided insights into the immune response, immune system development, and the effects of IL-2 treatments combined with radiotherapy. Blood samples were collected using anticoagulant tubes (with Heparin) from the cheeks of 3 mice/group on day 16 of the 344SQ-P tumor model. Information regarding the bioinformatics analysis has been previously delineated [20]. The raw TCR sequencing data was processed using MiXCR (version 3.0.13) with default parameters. Further bioinformatics analysis and data visualization was performed using the Immunarch package in R (version 4.0.1) (see detailed information in Supplementary Methods).

### Statistical analyses

Statistical analyses were done with GraphPad Prism. Tumor growth curves were compared using a two-way analysis of variance (ANOVA) with multiple comparisons. Unpaired *t* tests were used to compare differences between individual treatment groups. Survival rates were reported with the Kaplan–Meier curves and compared with log-rank tests. Statistical significance was defined as *P* ≤ 0.05.

## Results

### Optimizing the dosing *of alpha* subunit receptor-modified IL-2 cytokine treatment (RDB 1462) with Radiotherapy

**Fig. 1:**
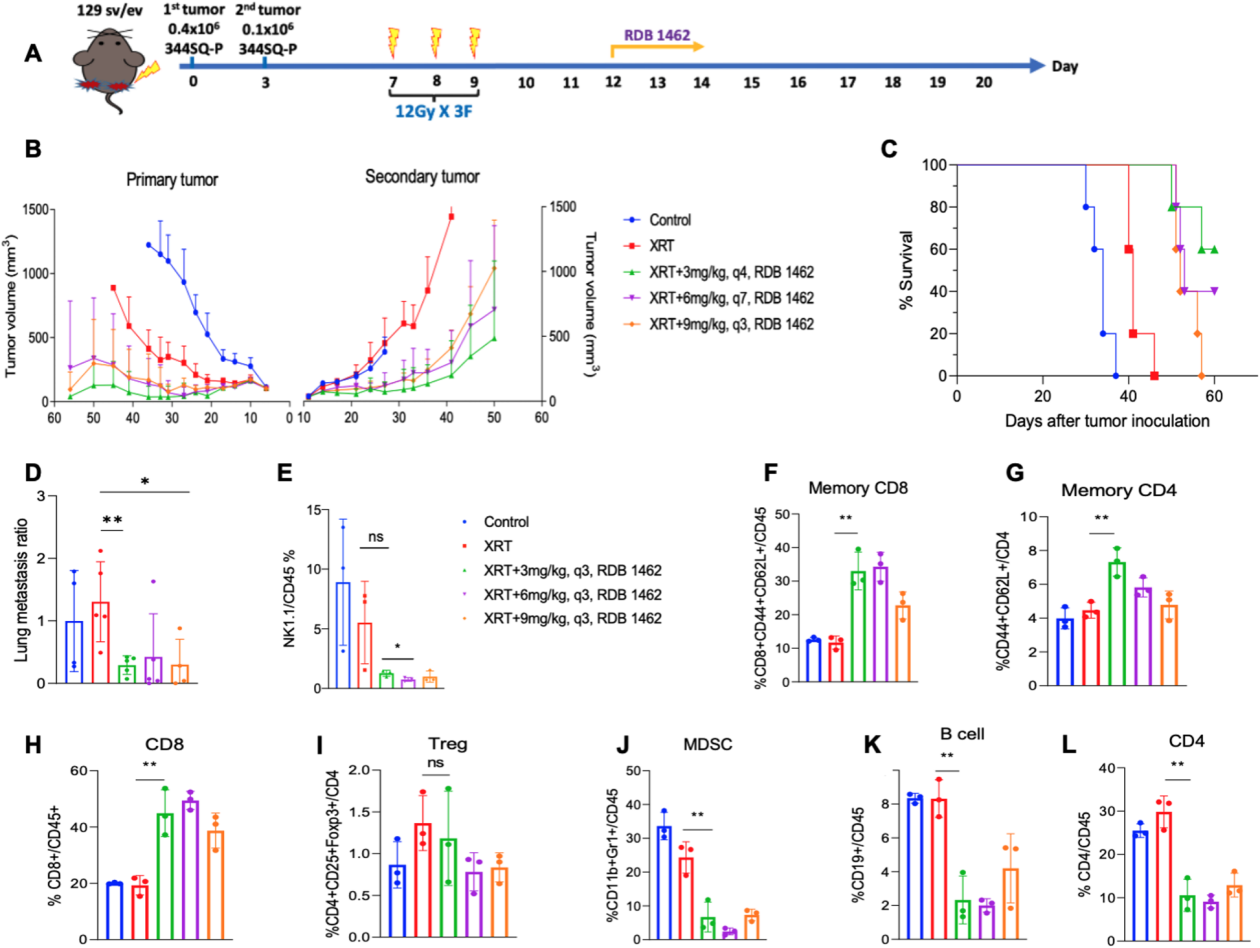
Effects of XRT and RDB 1462 on tumor growth and immune response. **A** 344SQ-P model establishment and treatment strategy. Mice received fractions of 12 Gy radiation starting on day seven post-implantation, followed by injections of different doses of RDB 1462-engineered IL-2 on day 12. **B** Tumor growth curves of primary and secondary tumors. **C** Survival curves for the five treatment groups. *N* = 5 mice/group and each experiment was repeated twice. Two-way ANOVA with multiple comparisons was used to analyze the tumor growth curves. Survival was plotted using the Kaplan–Meier method. **D** Lung metastasis for the five treatment groups. **E**-**L** Flow cytometry analysis of immune cells in blood harvested from different groups on day 24 (*n* = 5 mice/group), and each experiment was repeated twice. **E** Dual therapy with RDB 1462 led to a decrease in the NK1.1/CD45 percentage. **F**-**I** XRT combined with RDB 1462 enhanced memory CD4 + and CD8 + T cells, preferentially increased CD8 + T cell production, and suppressed Tregs. **J**-**L** Dual therapy reduced the numbers of MDSCs, B cells, and CD4 + T cells. *, *P* < 0.05; **, *P* < 0.01; ns, not significant, unpaired t test

In order to determine the optimal dosing of RBD1462 as to maximize therapeutic response when combined with high-dose RT, we evaluated several dosing schedules in a bilateral tumor model setting. 129/SvEv mice were subcutaneously inoculated in the right hind leg on day zero to establish the primary tumor, followed by the contralateral implantation of a secondary tumor three days later (0.4 and 0.1 × 10^6^ cells, respectively) with the mouse lung adenocarcinoma parental cell line 344SQ-P, which carries Kras^G12D^;p53R^172HDG^ mutations and was obtained from mice that recapitulate features of patients prone to metastatic lung cancer [21]. Bilateral tumor implantation in the lower extremities provides a strategic advantage for precise radiotherapy administration and facilitates accurate tumor measurement acquisition. On day 7 post primary tumor implantation, the primary tumor was irradiated with three fractions of 12 Gy, followed by the administration of different doses of RDB 1462-engineered IL-2 on day 12 (Fig. 1A). Our results indicate that radiation to the primary tumor site leads to an antitumor response beyond the irradiation field, impacting the secondary tumor site, and combining XRT with RDB 1462 notably reduces the primary tumor volume and controls the growth of the secondary tumor up to 50 days post-tumor implantation, with efficacy observed at 3 mg/kg doses (Fig. 1B, Supplementary Table 1). This outcome is attributed to a phenomenon known as the abscopal effect, where irradiation of a primary site with high-dose RT induces an immune-mediated response at secondary lesions outside the irradiation field, resulting in a significant reduction in the tumor volume. Notably, the tumor volume outcomes closely align with the percentage survival data for the experimental mice, demonstrating a substantial increase in survival rates, primarily evident in the XRT + RDB 1462 (3 mg/kg) group (Fig. 1C, Supplementary Table 2, Supplementary Fig. 2A). Furthermore, we observed a substantial reduction in lung metastasis ratios across all combination therapy groups, with the most pronounced effect observed in the XRT + RDB 1462 (3 mg/kg) group as compared to the XRT-treated group (*p*-value = 0.0087), or XRT-treated compared to the group receiving XRT + RDB 1462 (9 mg/kg) therapy (*p*-value = 0.029) (Fig. 1D). Surprisingly, the dual therapy, regardless of the dose of RDB 1462, led to a decrease in the NK1.1/CD45 percent (Fig. 1E), and we hypothesize this could be a transient result from the administration of RDB 1462 in which the engineered IL-2 could have some immunomodulatory effects that might indirectly affect NK cells numbers and activity. Remarkably, XRT combined with RDB 1462 resulted in the enhancement of memory CD8 + and CD4 + T cells (*p*-value = 0.021 and 0.007, respectively) (Fig. 1 F and G), and preferentially increased the production of CD8 + T cells (*p*-value = 0.008) (Fig. 1H), and suppressed the production of Tregs compared to the XRT-treated group (Fig. 1I), and reduced the numbers of myeloid-derived suppressor cells (MDSC) (*p*-value = 0.009) (Fig. 1J). In addition, the dual combination reduced the levels of B cells and CD4 + T-cells (*p*-value = 0.005 and 0.003, respectively) (Fig. 1 K and L), likely related to its role in differential binding to the IL-2 receptor. These events might increase the potential to improve the antitumor immune response. This study also highlighted the superiority of RDB 1462 over native IL-2 (RDB-1475) in controlling both primary and secondary tumors growth (*p*-value = 0.002 and < 0.0001, respectively) (Supplemental Fig. 1A) and enhancing the antitumoral immune response as observed in the increase in the percent in CD8 T cell populations (*p*-value = 0.002) (Supplemental Fig. 1B), and survival (Supplemental Fig. 1C). In summary, the combination of high-dose radiation therapy with RDB 1462-engineered IL-2 profoundly impacted tumor growth, metastasis, and the immune response in the experimental mouse model. The findings suggested that this combination therapy could be a promising approach for improving antitumor immune responses and potentially enhancing cancer treatment.

### Strategizing the sequencing and timing of optimal RDB 1462 dosage in conjunction with radiotherapy

**Fig. 2:**
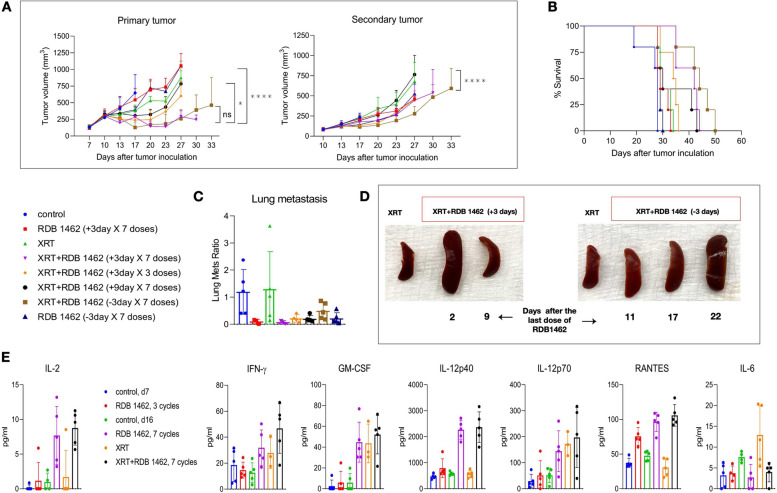
RDB 1462, administered three days before XRT, exhibits a profound anti-tumor effect. **A** Tumor growth curves of primary and secondary tumors, and comparing tumor volume on 30 days post-tumor implantation. **B** Survival curves for the eight treatment groups. **C** Lung metastasis for various treatment groups. **D** Spleens of mice from the indicated groups. RDB 1462 combined with XRT induced spleen enlargement due to enhanced immune cell accumulation. **E** Cytokine profiling of different groups. Blood samples were collected from various groups on day 7 and day 16. *N* = 5 mice/group, and each experiment was repeated twice. Persistent IL-2 levels were observed in mice treated with RDB 1462, alone or combined with XRT. Increased serum levels of IFN-γ, GM-CSF, IL-12, and RANTES in mice treated with RDB 1462 combined with XRT. Two-way ANOVA with multiple comparisons was used to analyze the tumor growth curves. Survival was plotted using the Kaplan–Meier method. *, *P* < 0.05; ****, *P* < 0.0001

We aimed to optimize the therapeutic use of RDB 1462 when combined with XRT to treat solid tumors. We conducted an extensive study into the timing of RDB 1462 administration, specifically assessing its impact when administered in seven cycles, with each cycle comprising three days before or after XRT (Fig. 2). Our findings revealed that initiating RDB 1462 administration three days before radiation therapy, repeated over seven cycles, yielded the most profound effects on primary and secondary tumors. This treatment approach substantially reduced tumor size and extended the survival of the experimental mice (all *p*-values < 0.05, Fig. 2A and B). Furthermore, RDB 1462, administered three days before XRT, was particularly effective in preventing tumor metastasis compared to the treatment of XRT alone (Fig. 2C). Also, this study showed that mice treated with RDB 1462 combined with XRT exhibited spleen enlargement compared to those treated with XRT alone (Fig. 2D); this is due to an enhanced therapeutic response attributed to the immunomodulatory effects of IL-2. IL-2 promotes the proliferation, differentiation, and activation of T-cells and NK cells. As a result, these immune cells tend to accumulate in lymphoid tissues, including the spleen.

In addition to assessing the structural changes in the spleen, we also examined the levels of IL-2 in the blood of treated mice. We observed persistent IL-2 levels in mice subjected to seven cycles of RDB 1462 treatment, whether administered alone or combined with XRT (Fig. 2E). Furthermore, we assessed the serum levels of IFN-γ and granulocyte–macrophage colony-stimulating factor (GM-CSF), IL-12, IL-6, and regulated upon activation, normal T cell expressed and presumably secreted (RANTES). Notably, mice treated with RDB 1462 combined with XRT exhibited increased levels of these molecules (Fig. 2E), which indicate in concert a continuous effect in chemotaxis and migration on T cell and monocytes (RANTES), and activation of T cells (Th1 cells) (IL-12), and lower levels in IL-6 might indicate the resolution of the acute phase in inflammatory response. These findings collectively illustrate that the combined therapy involving RDB 1462 and XRT, especially when administered in a specific timing regimen, exerts a substantial anti-tumor effect. This effect is mediated by the immunostimulatory properties of RDB 1462, resulting in increased immune cell activity, heightened cytokine levels, and enhanced immune responses, all of which contribute to the observed reduction in tumor size, extended survival, and prevention of metastasis.

### Enhancing the therapeutic efficacy with the triple combination of XRT + RDB 1462 + anti-PD1 for superior antitumor outcomes

**Fig. 3:**
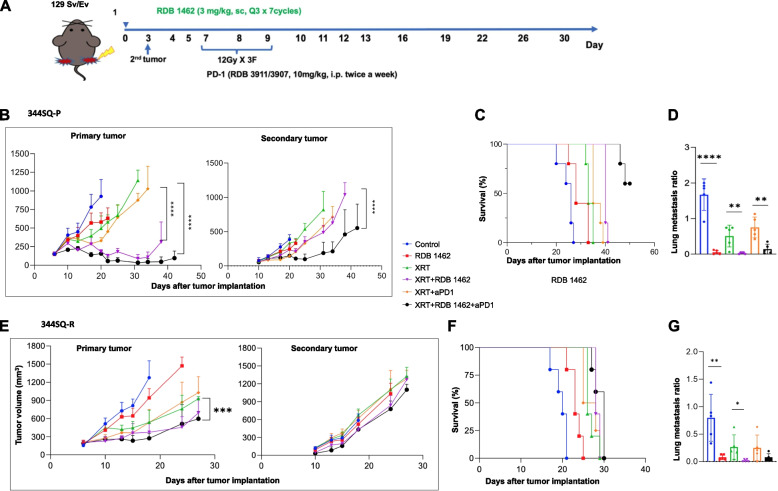
Triple therapy with RDB 1462, XRT, and αPD1 is a promising treatment approach. **A** 344SQ-P and 344SQ-R models establishment and treatment strategy. Mice were implanted with two tumors, one on each hind leg. RDB 1462 was administered three days before XRT, and repeated over seven cycles. Anti-PD1 was started on the same day with XRT, twice a week. **B** Growth curves of primary and secondary tumors in the indicated treatment groups in the 344SQ-P model. **C** Survival curves for the six treatment groups in the 344SQ-P model. **D** Lung metastasis for the indicated treatment groups in the 344SQ-P model. **E** Growth curves of primary and secondary tumors in the indicated treatment groups in the 344SQ-R model. **F** Survival curves for the indicated groups in the resistance model. **G** Lung metastasis for the indicated treatment groups in the resistance model. *N* = 5 mice/group and each experiment was repeated twice. Two-way ANOVA with multiple comparisons was used to analyze the tumor growth curves. Survival was plotted using the Kaplan–Meier method. *, *P* < 0.05; **, *P* < 0.01; ***, *P* < 0.001; ****, *P* < 0.0001

We utilized a two-tumor experimental model to evaluate the systemic anti-tumor efficacy of a triple therapy consisting of XRT, RDB 1462, and αPD1 therapy. The 129 Sv/Ev mice were implanted with the mouse lung adenocarcinoma parental cell line, 344SQ-P, bilaterally as detailed above. We also tested the combination treatment in a checkpoint resistant setting by utilizing the 344SQ-R cell line, an anti-PD1 resistant lung adenocarcinoma cell line. The tumor implantation schedule follows that of 344SQ-P cell line but through the inoculation of less cells (5 × 10^5^ cells for primary and 2.5 × 10^5^ cells for secondary). Three days after the primary tumor implantation, we administered RDB 1462 (3 mg/kg) (Fig. 3A). Subsequently, after an additional three-day interval, high-dose radiation therapy (XRT; 12 Gy x 3F) was administered to the primary tumor over three consecutive days. Following the three fractions of XRT, anti-PD1 therapy (10 mg/kg) was administered twice weekly (Fig. 3A). Similar to the previous findings with the dual combination of XRT + RDB1462, the triple combination of RDB 1462 + XRT + αPD1 led to a substantial reduction in the primary tumor volume and with sustained control of the secondary tumor growth in the parental model (Fig. 3B). Notably, the survival rate significantly improved with the triple therapy regimen compared to individual or dual therapies (Fig. 3C). Combining RDB 1462 with XRT alone or with XRT + αPD1 markedly decreased lung metastasis (Fig. 3D). Next, we tested the same therapeutic schema (Fig. 3A) in mice implanted with the 344SQ-R cell line. We observed a reduction of the abscopal effect even in the group receiving the triple therapy (Fig. 3E). However, the triple therapy (XRT + RDB 1462 + αPD1) and the dual therapy (XRT + RDB 1462) maintained a significant tumor control (all *p*-values < 0.05) than the monotherapies or XRT + αPD1 therapy (Fig. 3E). However, given the aggressiveness of this cell line, the mice succumbed to the secondary tumor burden at day 30 (Fig. 3F, Supplementary Fig. 2B), although the RDB 1462 alone or combined with XRT or XRT plus αPD1 therapy maintained a robust control in metastasis to the lung (Fig. 3G). This study highlights the remarkable antitumor efficacy of the triple therapy regimen comprising RDB 1462, high-dose XRT, and αPD1 therapy. This treatment approach effectively reduced primary and secondary tumor growth, improved survival rates, and exhibited promise in controlling tumor metastasis in the parental model, and a moderate effect in the anti-PD1 resistant model.

### Immunological cell profile modulations in primary and secondary tumor microenvironments and spleen post-treatment

**Fig. 4:**
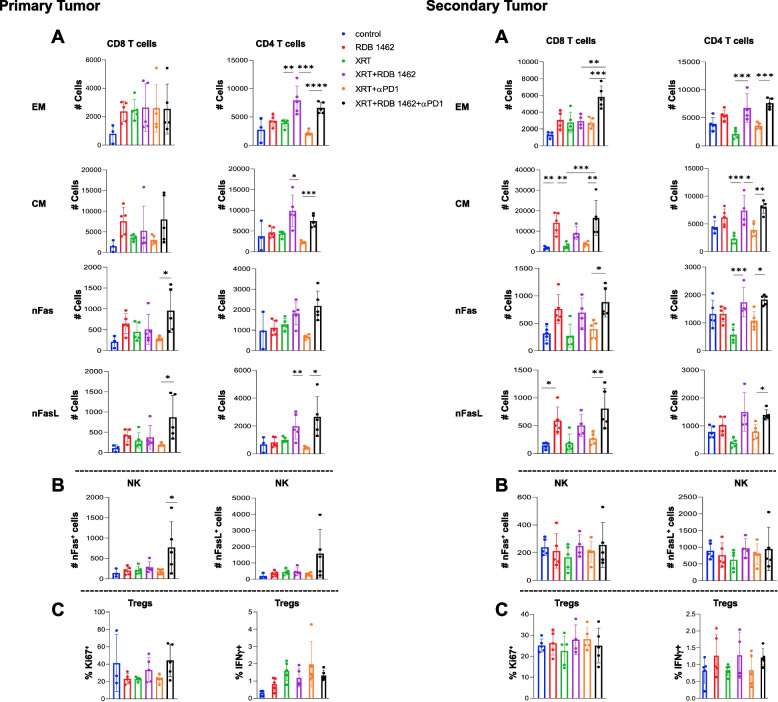
Triple therapy alters the tumor microenvironment by enhancing T cell function and promoting cell turnover. Flow cytometry analysis of immune cells in primary and secondary tumors from various treatment groups at 10 days after XRT. *N* = 5 mice/group and each experiment was repeated twice. The number of effector memory (EM), central memory (CM), Fas, and FasL in CD4 and CD8 T cells in primary (**A**) and secondary (**B**) tumors. The number of nFas-positive NK cells in the primary (**C**) and secondary (**D**) tumors. The percentage of Ki67 and IFN-γ positive regulatory T (Treg) cells in primary (**E**) and secondary (**F**) tumors across all treatments. *, *P* < 0.05; **, *P* < 0.01; ***, *P* < 0.001; ****, *P* < 0.0001, unpaired t test

In this study, our focus was to investigate the immune cell dynamics within primary and secondary tumors in mice subjected to various treatment regimens, including XRT in combination with RDB 1462, with or without anti-PD1 therapy. We employed specific markers, including CD45, CD3, CD4, CD8, CD44, and CD62L for T cells, as well as CD4, CD25, and Foxp3 for T regs, and CD49b, NK1.1 for NK cells, to identify and characterize the infiltrating immune cells in both primary and secondary tumors. Our findings suggest that the triple therapy, consisting of XRT, RDB 1462, and anti-PD1 therapy, led to notable alterations in the tumor microenvironment. Such alterations included the significant increase in the effector memory (EM) CD4 T cells in the primary and secondary tumors, and a significant increase of EM CD8 T cells in the secondary tumor, suggesting a heightened presence of T cells primed for immediate immune responses against the tumor. Central memory (CM) T cells play a pivotal role in establishing long-lasting immune responses, the triple combination therapy also enhanced the cell number of CM CD4 and CD8 T cells in both the primary and secondary tumors, as compared to XRT combined with anti-PD1 therapy, XRT alone, or control groups (Fig. 4 Primary and Secondary Tumors A).

Notably, the triple therapy also showed an enhancement in the expression of nFas and nFasL in CD8 and CD4 T cells in the primary tumor and secondary tumors, as compared to mice treated with XRT and anti-PD1 therapy, indicating a great turnover of these cells in which the promotion of programmed cell death may facilitate the replacement of unstable or exhausted T cells with healthier and more effective counterparts (Fig. 4 Primary and Secondary Tumors A). Similarly, mice treated with the triple therapy also showed increased levels of nFas and nFasL positive NK cells, likely indicating an enhanced turnover of these cells in the primary tumor but not in the secondary tumor (Fig. 4 Primary and Secondary Tumors B). This observation underscores the profound immunological alterations induced by the treatment, as nFas-positive cells are associated with immune regulation and apoptotic processes. Finally, we observed no significant differences in the proliferation of regulatory T cells, as assessed by the expression of Ki67, across all treatments in both primary and secondary tumors. Also, we did not see substantial differences in the number of IFN-γ + T regs in both primary and secondary tumors across treatments (Fig. 4 Primary and Secondary Tumors C).

### Transcriptomic profiling of lung adenocarcinoma samples under the influence of RDB 1462 combined with XRT

**Fig. 5:**
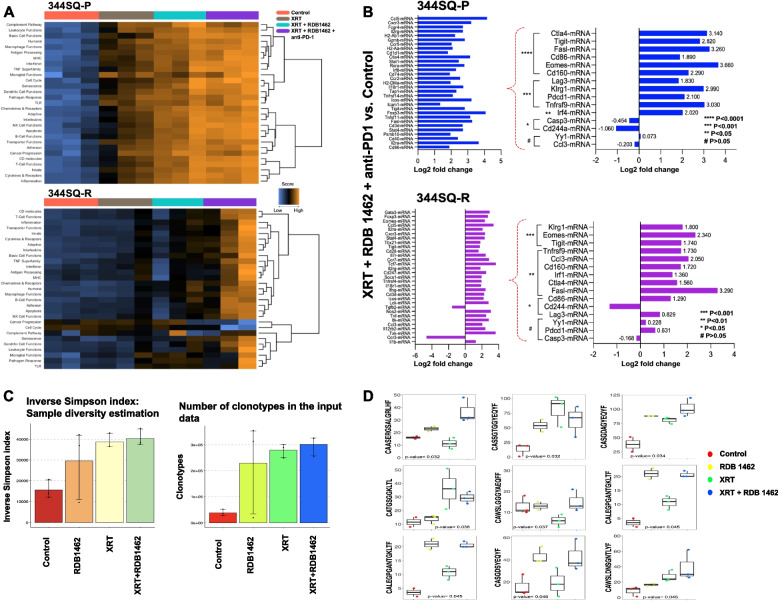
Triple therapy alters the gene expression profile of tumor-infiltrating immune cells and sophisticated immune regulation. **A** and **B** NanoString analysis of the tumor microenvironment. Secondary tumors in the 344SQ-P and 344SQ-R models were harvested at seven days post-XRT, followed by RNA extraction and NanoString immune profiling for 770 immune-related genes. **A** Heatmaps showing pathway score. Orange indicates high scores; blue indicates low scores. Scores are displayed on the same scale via a Z-transformation. **B** Specific genes with significant differences in the comparison of XRT + RDB 1462 + anti-PD1 and control in the 344SQ-P and 344SQ-R models. **C** and **D** TCR repertoire analysis. Blood samples were collected from the cheek of 3 mice/ group on day 16 in 344SQ-P tumor model. **C** The Simpson index (sample diversity) and the number of clonotypes for the indicated groups. D) Specific clonotypes were identified as increasing or appearing unique when treated with XRT or combined with RDB 1462

The study focused on understanding cancer-immune class-specific gene expression profiles in response to various therapeutic strategies. As previously stated, we used the two-tumor model (using parental 344SQ-P and the anti-PD1 resistant 344SQ-R, separately) with its respective implantation schedules, and therapies. The transcriptional analysis involved different experimental groups, encompassing a control, XRT-only treatment, XRT combined with RDB 1462, and the triple combination of XRT, RDB 1462, and anti-PD1 therapy. We observed distinct transcriptional profiles in immune functional pathways when comparing the 344SQ-R tumor model, known for its resistance to anti-PD1, with its parental control, 344SQ-P. The parental control exhibited greater sensitivity to changes induced by XRT, its combination with RDB 1462, or the triple combination involving anti-PD1 therapy (Fig. 5A 344SQ-P). In contrast, the resistant tumor model primarily responded to the triple combination XRT + RDB 1462 + anti-PD1 (Fig. 5A 344SQ-R). This unique response of the resistant tumor is particularly noteworthy since it tends to be more aggressive, with extensive metastatic potential.

To gain deeper insights into the components driving the antitumor immune response, we conducted a comprehensive transcriptional analysis of tumor-infiltrating immune cells obtained from mice subjected to the triple therapy XRT + RDB 1462 + anti-PD1. The results showed a significant increase of 1.5-fold or more in the expression of several key genes in mice implanted with the parental cell line, including Ctla-4, Tigit, Lag3, FasL, Cd86 Eomes, Klrg1, Tnfrsf9 (4-1BB), and Cd160 (Fig. 5B 344SQ-P). This gene expression profile conveys a compelling description of the dynamic processes unfolding within the antitumor immune milieu. Eomes, Klrg1, Tnfrsf9 (4-1BB), and Cd160 upregulation suggests an amplified immune response [22–27]. Simultaneously, the increased expression of genes like Ctla-4 and Tigit suggests an active effort within these immune cells to modulate their responses, as these genes play a crucial role in immune checkpoint regulation, indicating that the immune system tries to fine-tune its responses. The increased expression of FasL in these immune cells implies the promotion of programmed cell death of immune cells. This mechanism may facilitate the replacement of unstable or exhausted immune cells with healthier and more effective counterparts [28]. Immune cells obtained from mice implanted with the anti-PD1 resistant cell line (344SQ-R) and treated with the triple therapy displayed a similar more than 1.5-fold increase in genes such as Klrg1, Eomes, Tigit, Tnfrsf9, Ccl3, Cd160, Ctla-4, and FasL (Fig. 5B 344SQ-R). This suggests that the triple therapy induces mostly comparable gene expression patterns in both tumor models with the exception of some notable genes of interest dissected below.

The heightened expression of FasL in both tumor models underlines the potential influence of the triple therapy in inducing an active turnover from exhausted to healthier tumor-infiltrating immune cells. In contrast, we observed increased expression of Ccl3 in the anti-PD1 resistant tumor model compared to the parental model, indicating enhanced recruitment of immune cells and an adequate pro-inflammatory response in the resistant tumors. Another intriguing observation was the differential expression of PD1 in the two tumor models. While the parental model exhibited a 2.1-fold increase in PD1 expression, the resistant model showed only a 0.63-fold increase; this high-lights varying PD1 expression patterns between the tumor models. This comprehensive analysis revealed a complex interplay of gene expression changes triggered by the combined therapy, reflecting enhanced antitumor activity and a sophisticated immune regulation mechanism to achieve an optimal and balanced immune response. These findings offer valuable insights into the intricate dynamics of tumor-immune interactions in the context of different therapeutic approaches.

Next, to understand the extent to whether the therapeutic regimes had any impact in the generation of a distinctive TCR repertoire, we performed TCR sequencing in T lymphocyte samples from the control, RDB 1462, XRT, and XRT + RDB 1462 groups. The inverse Simpson index for diversity estimation and the analysis of the number of clonotypes indicate that XRT or XRT combined with RDB 1462 increased the Simpson index and the number of clonotypes (Fig. 5C). We have identified at least eight different clonotypes increasing under the use XRT or combined with RDB 14621. One clonotype (CALEGPGANTGKLTF) seems to be unique to the use of RDB 1462 (Fig. 5D). We are further analyzing the relevance of these clonotypes under these treatment conditions.

## Discussion

We demonstrated the potential of combining RDB 1462, high-dose radiation therapy (XRT), and αPD1 therapy in two-tumor mouse models. This triple therapy regimen exhibited remarkable antitumor efficacy, significantly reducing primary and secondary tumor growth, improving survival rates, and effectively controlling tumor metastasis in the parental model. The synergistic effects of this combination are attributed to the unique properties of its components: A) RDB 1462: Enhances the activation and proliferation of tumor-specific T cells; B) XRT: Controls primary tumor growth and triggers the release of tumor antigens that prime the immune system’s specificity towards secondary tumor sites; C) αPD1 therapy: Blocks the PD1/PD-L1 inhibitory pathway, unleashing T cell activity against tumor cells. The optimal timing of RDB 1462 administration, when combined with XRT, further amplified the antitumor effects. Initiating RDB 1462 administration three days before XRT, repeated over seven cycles, yielded the most pronounced antitumor effects. This specific timing regimen allowed RDB 1462 to prime the immune system before radiation therapy, maximizing its immunomodulatory effects. Specifically, our results show that RDB 1462 induces IL-2-mediated effects that synergize with the immunostimulatory impact of radiation therapy. Our initial assessments focused on the synergistic potential of combining XRT with RDB 1462 to achieve an enhanced inhibitory effect on tumor growth and extend overall survival. Interestingly, we observed that the 3 mg/kg q4 dose of RDB 1462 demonstrated superior effectiveness to higher doses of 6 mg/kg q7 and 9 mg/kg q3. Notably, recent research has emphasized the significance of combining immunogenic XRT with IL-2c (IL-2 complexes), specifically targeting the low-affinity IL-2 receptor. This strategic approach has been shown to produce a synergistic and more potent therapeutic effect compared to combinations involving uncomplexed IL-2, underscoring the potential of our chosen treatment regimen [29].

Combining XRT and IL2c led to a notable expansion of CD8 + T cells and increased spleen weight. Also, within the combination treatment groups, there was a significant elevation in the levels of memory CD8 and CD4 cells compared to the XRT control group. Conversely, there was a reduction in the levels of immunosuppressive Treg cells, MDSC, CD4, and B cells within the combination therapy groups compared to the XRT control. Our study demonstrates a synergistic effect between XRT applied to a single tumor (abscopal effect) and systemic therapy involving RDB 1462, resulting in a substantial increase in tumor control and survival of mice bearing bilateral tumors, in contrast to the outcomes of XRT or RDB 1462 monotherapies. This combination therapy enhanced the magnitude and effector function of tumor-specific CD8 + T cell responses and facilitated the migration of these T cells to both irradiated and distant, unirradiated tumors. Previous reports indicated that applying RT to a single tumor with NKTR-214 systemic therapy leads to increased tumor control of mice bearing bilateral tumors, outperforming the effects of RT or NKTR-214 monotherapies [30].

This combination therapy amplified the magnitude and effector function of tumor-specific CD8 + T cell responses and enhanced the trafficking of these T cells to both irradiated and distant, unirradiated tumors. In this study, we noticed that administering RDB 1462 at a dose of 3 mg/kg before radiation therapy provided more substantial support for antitumor efficacy and led to a higher percentage of animal survival than post-administration of RDB 1462. This effect might be attributed to the sustained sensitization of CD8 + T and NK cells in the tumor microenvironment to IL-2 due to RDB 1462, which amplifies the immunogenic effects of RT.

Additionally, levels of critical immune markers, including IL-2, IFN-γ, and GM-CSF, were elevated in groups treated with RDB 1462 and the combination of XRT and RDB 1462. These findings align with reports indicating that radiotherapy and anti-PD1 treatment led to a marked reduction in tumor volume in mice [31]. Another study further demonstrates that the effectiveness of anti-PD1 therapy relies on the signaling of IL-2, and the combination of low-affinity IL-2 with anti-PD1 amplifies the antitumor response by promoting the expansion of CD8 + T cells and suppressing Treg activity [32]. Likewise, a recombinant IL-2, which includes a tumor-targeting antibody (Ab) and a super mutant IL-2 (sumIL-2), referred to as Ab-sumIL2, substantially boosts antitumor efficacy by precisely targeting tumors and binding specifically to cytotoxic T lymphocytes (CTLs) [33]. In our study, the triple therapy regimen, which includes XRT, Anti-PD1, and RDB 1462, exhibited a remarkable suppression of tumor growth. Primary tumor suppression was highly significant in the XRT + Anti-PD1 + RDB 1462, and XRT + RDB 1462 groups and the growth rate of secondary tumors was diminished, with substantial improvement in survival rates in the triple combination group. The lung metastasis ratio also substantially reduced the RDB 1462, XRT + RDB 1462, and XRT + Anti-PD1 + RDB 1462 groups. A recent study corroborated these findings, underscoring the antitumor effectiveness of targeting PD1 and IL-2Rβγ in combination with radiation therapy to impede the growth and metastasis of pancreatic cancer [11].

Also, our study investigated the effects of different therapeutic regimens on the immune cell profile of primary and secondary tumors in mice. The results showed the triple therapy significantly altered the immune cell dynamics within primary and secondary tumors. The number of EM CD4 and CD8 T cells increased substantially, suggesting a heightened presence of T cells primed for immediate immune responses against the tumor. Additionally, triple therapy expanded CM T cells, playing a crucial role in establishing long-lasting immune responses. The triple therapy induced the expression of Fas and Fasl in CD8 and CD4 T cells, potentially facilitating the replacement of unstable or exhausted T cells with healthier and more effective counterparts [34]. This turnover of immune cells is crucial for maintaining a robust antitumor response.

Transcriptional analysis revealed distinct gene expression profiles in immune functional pathways when comparing the PD1-resistant tumor model with its parental control. The triple therapy demonstrated the most significant impact on transcriptional profiles, particularly in the resistant tumor model, suggesting that triple therapy may effectively overcome resistance mechanisms and enhance treatment outcomes in patients with various types of cancer. In particular, the triple therapy induced a substantial increase in the expression of genes involved in immune checkpoint regulation (Ctla-4, Tigit, Lag3), immune activation (Eomes, Klrg1, Tnfrsf9, Cd160), and programmed cell death (FasL); these changes reflect the enhanced antitumor activity and sophisticated immune regulation mechanism induced by this therapy.

Treatment with XRT or XRT combined with RDB 1462 increased TCR repertoire diversity and the number of clonotypes, suggesting that these treatments effectively modulate the tumor microenvironment, leading to the expansion of tumor-specific T cell clones. Intriguingly, both XRT and XRT combined with RDB 1462 demonstrated an increase in the Simpson index and the number of clonotypes, suggesting an enhanced diversity of the TCR repertoire. This observation warrants further investigation to elucidate the potential implications of this repertoire expansion in the context of antitumor immunity. Notably, identifying a unique clonotype (CALEGPGANTGKLTF) associated explicitly with RDB 1462 treatment highlights the potential of this combination therapy in shaping the TCR repertoire. Ongoing studies aim to unravel the functional significance of these distinct clonotypes and their contribution to the overall antitumor response. Overall, the findings of these studies highlight the remarkable potential of the triple therapy regimen comprising RDB 1462, high-dose XRT, and αPD1 therapy as an effective cancer immunotherapy approach. The synergistic effects of this combination, its ability to overcome resistance mechanisms, and the observed alterations in immune cell dynamics and transcriptional profiles suggest that this therapy holds promise for improving treatment outcomes in patients with various types of cancer. Further clinical studies are warranted to evaluate the safety and efficacy of this triple therapy in human patients.

## Supplementary Information

Supplementary Material 1

Supplementary Material 2

Supplementary Material 3

## Data Availability

Not applicable.
