# Correction: Novel engineered IL-2 Nemvaleukin alfa combined with PD1 checkpoint blockade enhances the systemic anti-tumor responses of radiation therapy

**DOI:** 10.1186/s13046-024-03183-9

**Published:** 2024-09-10

**Authors:** Kewen He, Nahum Puebla-Osorio, Hampartsoum B. Barsoumian, Duygu Sezen, Zahid Rafq, Thomas S. Riad, Yun Hu, Ailing Huang, Tifany A. Voss, Claudia S. Kettlun Leyton, Lily Jae Schuda, Ethan Hsu, Joshua Heiber, Maria-Angelica Cortez, James W. Welsh

**Affiliations:** 1 Department of Radiation Oncology, Shandong First Medical University and Shandong Academy of Medical Sciences, Shandong Cancer Hospital and Institute, Jinan, Shandong China; 2 Department of Radiation Oncology, Division of Radiation Oncology, The University of Texas MD Anderson Cancer Center, Houston, TX USA; 3 Department of Radiation Oncology, Koç University School of Medicine, Istanbul, Turkey; 4 Mural Oncology PLC, Waltham, MA USA

**Correction: J Exp Clin Cancer Res 43, 251 (2024)**

10.1186/s13046-024-03165-x

Following publication of the original article [1], the authors found an error in the affiliation of the 5th author, Zahid Rafiq. He was mistakenly assign to Affiliation 1. The details are given below:

**Incorrect affiliation**:

^1^ Department of Radiation Oncology, Shandong First Medical University and Shandong Academy of Medical Sciences, Shandong Cancer Hospital and Institute, Jinan, Shandong, China.

**Correct affiliation**:

^2^ Department of Radiation Oncology, Division of Radiation Oncology, The University of Texas MD Anderson Cancer Center, Houston, TX, United States.

This correction does not affect the overall result or conclusion of the article. The original article [1] has been corrected.
