# Supplementary material for: Novel engineered IL-2 Nemvaleukin alfa combined with PD1 checkpoint blockade enhances the systemic anti-tumor responses of radiation therapy
Source: J Exp Clin Cancer Res. 2024 Sep 2;43:251. doi: 10.1186/s13046-024-03165-x (PMC11367833; doi:10.1186/s13046-024-03165-x)
Supplement: Supplementary file 1 — Supplementary Material 1 [file 13046_2024_3165_MOESM1_ESM.pptx]

## Slide 1
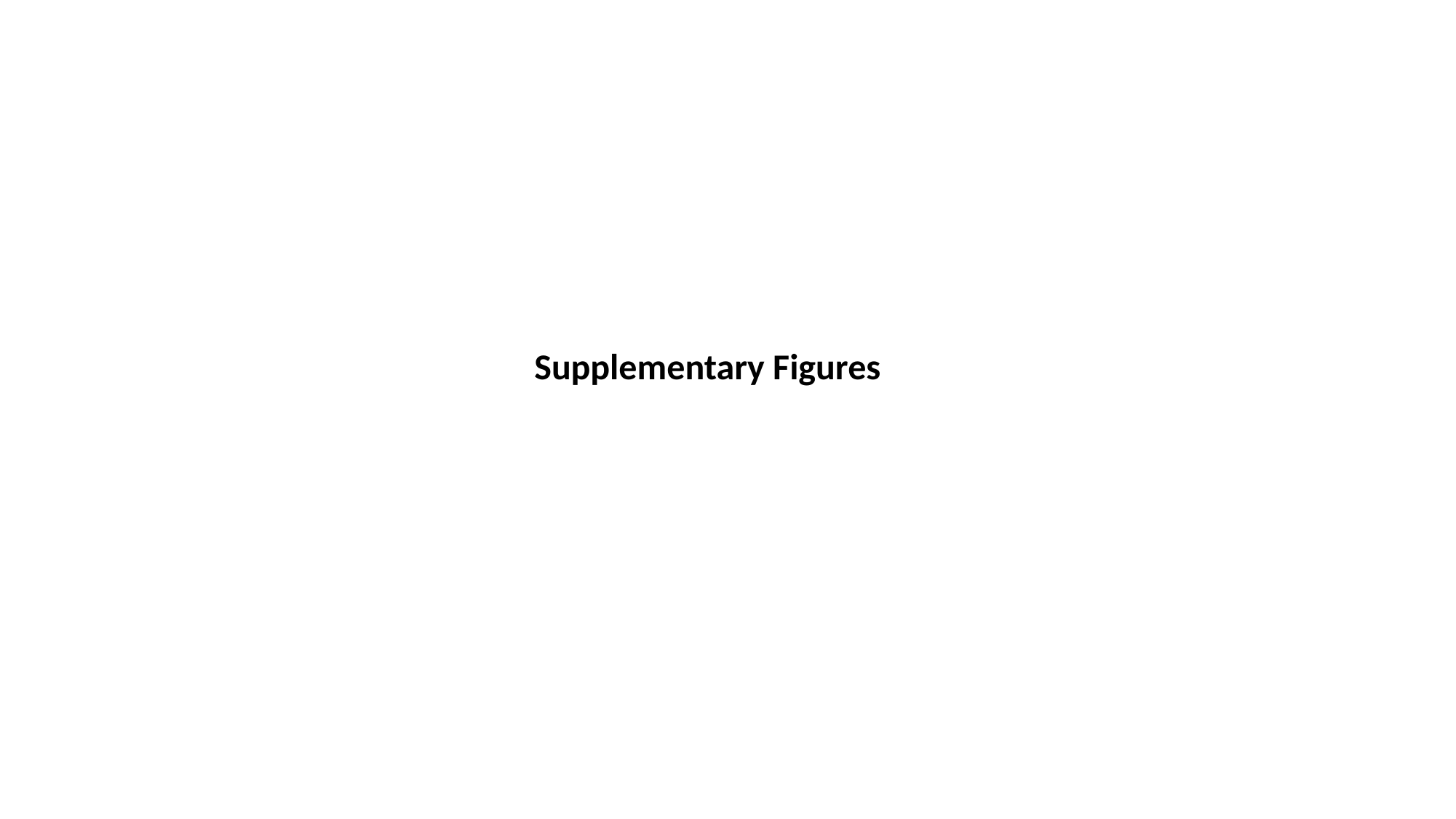

Supplementary Figures

## Slide 2
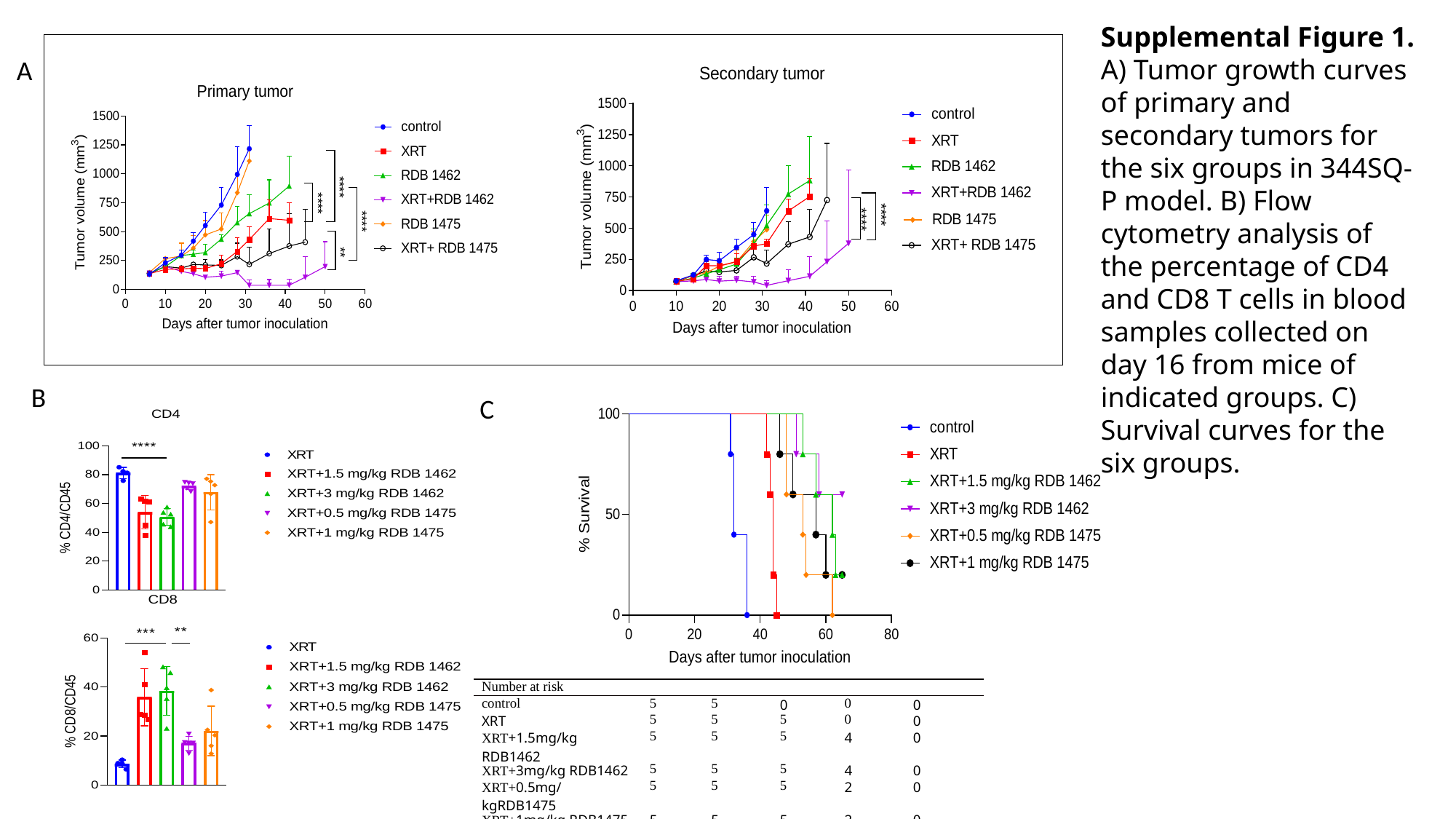

Supplemental Figure 1. A) Tumor growth curves of primary and secondary tumors for the six groups in 344SQ-P model. B) Flow cytometry analysis of the percentage of CD4 and CD8 T cells in blood samples collected on day 16 from mice of indicated groups. C) Survival curves for the six groups.
A
B
C
| Number at risk | | | | | |
| --- | --- | --- | --- | --- | --- |
| control | 5 | 5 | 0 | 0 | 0 |
| XRT | 5 | 5 | 5 | 0 | 0 |
| XRT+1.5mg/kg RDB1462 | 5 | 5 | 5 | 4 | 0 |
| XRT+3mg/kg RDB1462 | 5 | 5 | 5 | 4 | 0 |
| XRT+0.5mg/kgRDB1475 | 5 | 5 | 5 | 2 | 0 |
| XRT+1mg/kg RDB1475 | 5 | 5 | 5 | 2 | 0 |

## Slide 3
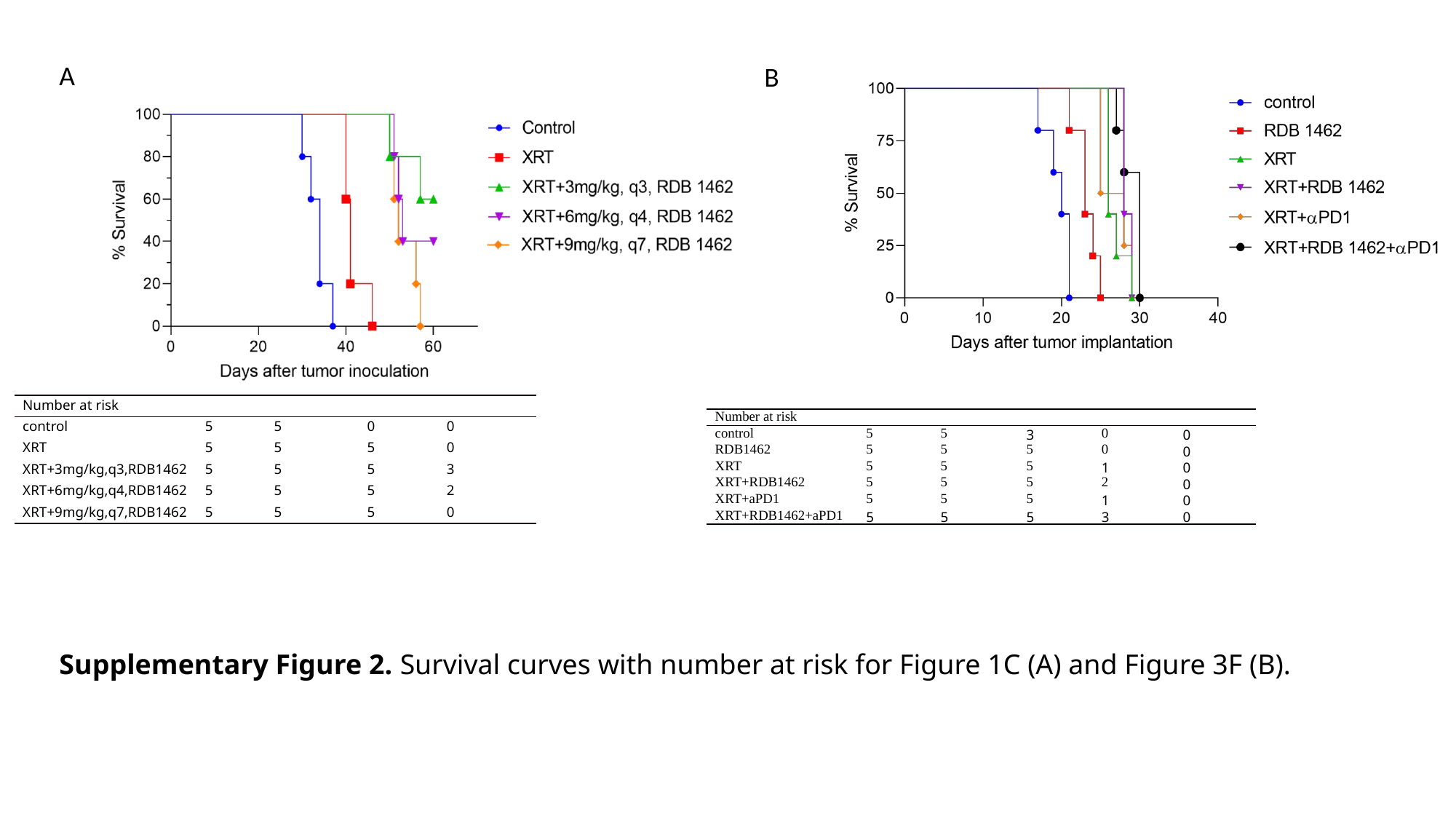

A
B
| Number at risk | | | | |
| --- | --- | --- | --- | --- |
| control | 5 | 5 | 0 | 0 |
| XRT | 5 | 5 | 5 | 0 |
| XRT+3mg/kg,q3,RDB1462 | 5 | 5 | 5 | 3 |
| XRT+6mg/kg,q4,RDB1462 | 5 | 5 | 5 | 2 |
| XRT+9mg/kg,q7,RDB1462 | 5 | 5 | 5 | 0 |
| Number at risk | | | | | |
| --- | --- | --- | --- | --- | --- |
| control | 5 | 5 | 3 | 0 | 0 |
| RDB1462 | 5 | 5 | 5 | 0 | 0 |
| XRT | 5 | 5 | 5 | 1 | 0 |
| XRT+RDB1462 | 5 | 5 | 5 | 2 | 0 |
| XRT+aPD1 | 5 | 5 | 5 | 1 | 0 |
| XRT+RDB1462+aPD1 | 5 | 5 | 5 | 3 | 0 |
Supplementary Figure 2. Survival curves with number at risk for Figure 1C (A) and Figure 3F (B).
