# Supplementary material for: Novel engineered IL-2 Nemvaleukin alfa combined with PD1 checkpoint blockade enhances the systemic anti-tumor responses of radiation therapy
Source: J Exp Clin Cancer Res. 2024 Sep 2;43:251. doi: 10.1186/s13046-024-03165-x (PMC11367833; doi:10.1186/s13046-024-03165-x)
Supplement: Supplementary file 2 — Supplementary Material 2 [file 13046_2024_3165_MOESM2_ESM.docx]

**Supplementary Methods**

**Detailed information for Nanostring**

RNA samples were extracted from Histopaque-enriched TILs (3 mice/group, two-tumor model of LLC) by an RNeasy Mini Kit (Qiagen) according to the manufacturer’s protocol. The RNA samples were submitted to the Advanced Technology Genomics Core at MDACC for NanoString analysis. Expression profiling was performed on the nCounter FLEX Instrument using nCounter Mouse PanCancer Immune Profiling panel (NanoString Technologies). Briefly, purified RNA was quantitated using the Qubit system (Life Technologies) and quality checked using NanoDrop One (Thermo Fisher Scientific) and TapeStation 4200 (Agilent). 100-150 ng, depending on the amount of degradation, of RNA were hybridized to gene-specific fluorescent-labeled probes. The hybridized products were then purified on the nCounter Prep Station. The fluorescent-labeled products were then scanned on the nCounter Digital Analyzer, and the nCounter Mouse PanCancer Immune Profiling panel was used and included 770 genes (https://nanostring.com/products/ncounter- assays-panels/oncology/). No custom probes were added for either panel. Data from the NanoString nCounter System were normalized to the internal positive controls and housekeeping genes using the recommended settings in the nSolver Software Normalization Module (NanoString Technologies). Normalized data were exported, and differential expression analysis was performed using a linear model method with the limma package for the R programming language. Cell typing and pathway analysis, and genes differentially expressed in each treatment were identified from the expression data of 770 genes by nSolver. Analysis and normalization of the raw NanoString data were done with nSolver Software v4.0 and nCounter Advanced Analysis (NanoString Technologies).

**Cytokine profiling**

We also analyzed serum pro-inflammatory cytokine markers to assess the systemic immune response and the presence of key cytokines associated with inflammation.

Cytokine analysis was conducted using a magnetic bead based multiplex assay system, with detection and quantification of multiple cytokines from a single sample of plasma (a mouse cytokine 23-plex assay, Bio-Plex Pro Mouse Cytokine 23-plex #M60009RDPD). In this test, 23 cytokines were simultaneously quantified that included eotaxin, granulocyte colony-stimulating factor (G-CSF), granulocyte-macrophage colony-stimulating factor (GM-CSF), interferon (IFN)-γ, interleukin (IL)-1β, IL-1α, IL-2, IL-3, IL-4, IL-5, IL-6, IL-9, IL-10, IL-12p40, IL-12p70, IL-13, IL-17A, KC, monocyte chemotactic protein (MCP)-1, macrophage inflammatory protein (MIP)-1α, MIP-1β, regulated on activation normal T cell expressed and secreted (RANTES), tumor necrosis factor (TNF)-α. All standards, reagents, and samples were prepared according to the manufacturer’s instructions. Blood samples were collected from the cheek of 5 mice/ group on day 16 and centrifuged for 20 minutes at 2000×g. Serums were collected and then diluted by 1:2 before loading and incubating with detection antibodies and SA-PE. The 96-well plate was read by Bio-Plex MAGPIX System. Standard curves for each analyte were generated using standards provided by the manufacturer.

**TCR repertoire and Bioinformatics analysis**

The murine 344SQ-P tumor model was used for assessing T-cell density and clonality. T-cell density was measured in the blood by RNA content. Blood samples were collected using anticoagulant tubes (with Heparin) from the cheek of 3 mice/ group on day 16. Extracted RNA from blood by Qiagen RNeasy Mini kit (catalog no.74106). The raw TCR sequencing data was processed using MiXCR (version 3.0.13) with default parameters. In brief, the raw reads were aligned to the *Mus Musculus* reference TCR genes based on the ImMunoGeneTics database (IMGT). Then, the aligned reads were assembled to construct the CDR3 (Complementarity-determining region 3). Finally, the MiXCR reported the clonotypes of each sample, in which the unique clonotype was defined as the unique CDR3 amino acid sequences and V-J segments genes. Further bioinformatics analysis and data visualization was performed using the Immunarch package in R (version 4.0.1). The clonality was defined as the Pielou’s evenness index, which was calculated using the formula $J= -\frac{\sum_{i=1}^{R} (p_{i}\times{log}_{2}p_{i})}{{log}_{2}R}$, where $p_{i}$ is the frequency/proportion of clone $i$ for a sample with a total of $R$ unique CDR3 sequences. The diversity of the repertories was measured using the Inverse Simpson index, $\frac{1}{\lambda}=\frac{1}{\sum_{i=1}^{R} p_{i}^{2}}$. The circlize package was used to generate the circos plot of each sample regarding V-J usage.
