# Supplementary material for: Novel engineered IL-2 Nemvaleukin alfa combined with PD1 checkpoint blockade enhances the systemic anti-tumor responses of radiation therapy
Source: J Exp Clin Cancer Res. 2024 Sep 2;43:251. doi: 10.1186/s13046-024-03165-x (PMC11367833; doi:10.1186/s13046-024-03165-x)
Supplement: Supplementary file 3 — Supplementary Material 3 [file 13046_2024_3165_MOESM3_ESM.docx]

**Supplementary Table 1. Multiple comparisons of primary and secondary tumors in different treatment groups**

|  | **Primary tumors** | | **Secondary tumors** | |
| --- | --- | --- | --- | --- |
|  | Mean (mm^3^) | *P* Value | Mean (mm^3^) | *P* Value |
| Control vs. XRT | 666.1 vs. 318.6 | <0.0001 | 291.1 vs. 556.6 | 0.7552 |
| Control vs. XRT+3mg/kg, q3, RDB 1462 | 666.1 vs. 82.33 | <0.0001 | 291.1 vs. 146.6 | <0.0001 |
| Control vs. XRT+6mg/kg, q4, RDB 1462 | 666.1 vs. 149.6 | <0.0001 | 291.1 vs. 228.1 | <0.0001 |
| Control vs. XRT+9mg/kg, q7, RDB 1462 | 666.1 vs. 148.6 | <0.0001 | 291.1 vs. 266.9 | <0.0001 |
| XRT vs. XRT+3mg/kg, q3, RDB 1462 | 318.6 vs. 82.33 | <0.0001 | 556.6 vs. 146.6 | <0.0001 |
| XRT vs. XRT+6mg/kg, q4, RDB 1462 | 318.6 vs. 149.6 | <0.0001 | 556.6 vs. 228.1 | <0.0001 |
| XRT vs. XRT+9mg/kg, q7, RDB 1462 | 318.6 vs. 148.6 | <0.0001 | 556.6 vs. 266.9 | <0.0001 |
| XRT+3mg/kg, q3, RDB 1462 vs. XRT+6mg/kg, q4, RDB 1462 | 82.33 vs. 149.6 | 0.0396 | 146.6 vs. 228.1 | 0.5482 |
| XRT+3mg/kg, q3, RDB 1462 vs. XRT+9mg/kg, q7, RDB 1462 | 82.33 vs. 148.6 | 0.0434 | 146.6 vs. 266.9 | 0.0001 |
| XRT+6mg/kg, q4, RDB 1462 vs. XRT+9mg/kg, q7, RDB 1462 | 149.6 vs. 148.6 | 0.9994 | 228.1 vs. 266.9 | 0.0071 |

**Supplementary Table 2. Survival comparison for mice with different treatment**

|  | Control | XRT | XRT+3mg/kg, q3, RDB 1462 | XRT+6mg/kg, q4, RDB 1462 | XRT+9mg/kg, q7, RDB 1462 |
| --- | --- | --- | --- | --- | --- |
| Median survival (days) | 34 | 41 | Undefined | 53 | 52 |
| Log-rank *P* value <0.0001 | | | | | |
